# Caspases in the Developing Central Nervous System: Apoptosis and Beyond

**DOI:** 10.3389/fcell.2021.702404

**Published:** 2021-07-16

**Authors:** Trang Thi Minh Nguyen, Germain Gillet, Nikolay Popgeorgiev

**Affiliations:** ^1^Centre de Recherche en Cancérologie de Lyon, U1052 INSERM, UMR CNRS 5286, Centre Léon Bérard, Université Claude Bernard Lyon 1, Lyon, France; ^2^Hospices Civils de Lyon, Laboratoire d’Anatomie et Cytologie Pathologiques, Centre Hospitalier Lyon Sud, Pierre Bénite, France

**Keywords:** caspases, mitochondria, central nervous system, apoptosis, embryonic development

## Abstract

The caspase family of cysteine proteases represents the executioners of programmed cell death (PCD) type I or apoptosis. For years, caspases have been known for their critical roles in shaping embryonic structures, including the development of the central nervous system (CNS). Interestingly, recent findings have suggested that aside from their roles in eliminating unnecessary neural cells, caspases are also implicated in other neurodevelopmental processes such as axon guidance, synapse formation, axon pruning, and synaptic functions. These results raise the question as to how neurons regulate this decision-making, leading either to cell death or to proper development and differentiation. This review highlights current knowledge on apoptotic and non-apoptotic functions of caspases in the developing CNS. We also discuss the molecular factors involved in the regulation of caspase-mediated roles, emphasizing the mitochondrial pathway of cell death.

## Introduction

Apoptosis is considered to be a programmed cell death (PCD) through which a multicellular organism removes damaged cells without affecting neighboring cells (Wickman et al., 2012). Apoptotic cells exhibit specific characteristics, including cell shrinkage, chromatin condensation and fragmentation, and cell membrane blebbing, followed by apoptotic body formation (Taylor et al., 2008). Shreds of evidence suggest that apoptosis, which is considered to be a self-destructive program, may also take place in healthy cells to ensure the daily functions of tissues and organs (Suzanne and Steller, 2013; Shalini et al., 2015).

The central nervous system (CNS), including the brain and spinal cord, is a complex structure that begins to appear at early stages of embryonic development. The first significant event is the formation of the neural tube from the neural plate during primary and secondary neurulation. During primary neurulation, a portion of dorsal ectoderm specifies into neural plate. Neural plate cells can be distinguished from surrounding non-neural cells by their elongated morphology. Shortly after its establishment, neural plate border thickens and rises up to create a U-shape groove. The two lateral sides of the neural groove continue to bend toward each other until their edges meet and merge to form the neural tube. Secondary neurulation then begins at the caudal end. In this latter process, that takes place in chicken and some mammalian embryos, mesenchymal cells gather to form the medullary cord under the ectoderm layer. In the next step, the medullary cord is reshaped to create a hollow cavity (Smith and Schoenwolf, 1997; Gilbert, 2000).

From a simple straight structure at the beginning, the anterior part of the neural tube expands to form three primary vesicles: prosencephalon (forebrain), mesencephalon (midbrain), and rhombencephalon (hindbrain). These latter vesicles continue to subdivide and ultimately form the different regions of the brain (Smith and Schoenwolf, 1997; Stiles and Jernigan, 2010). Concurrently, neural progenitors, derived from the neuroepithelial cells of the ectoderm, differentiate into neurons and glial cells (Götz and Huttner, 2005; Paridaen and Huttner, 2014). Neurons and glial cells are organized into layers (cortex) or clusters (nuclei). From the luminal germinal neuroepithelium, newborn cells migrate outward to form layers at the marginal zone (Ayala et al., 2007). The most recently born neurons migrate through the previous cell layers to give rise to the more superficial regions.

Apoptosis is known to play essential roles in the development of the CNS. Indeed, cell death events have been largely described in the brain of vertebrate embryos, whereas experimental suppression of apoptosis in the developing embryo mainly results in deleterious malformations in the nervous system (Yamaguchi and Miura, 2015).

Caspases, a family of intracellular proteases considered to be the executioners of apoptosis, have been highlighted for their role in the development of the CNS (Yuan and Yankner, 2000). Indeed, by acting as cell death accelerators, they precisely delineate the size of cell populations in the developing embryo, including in the brain and spinal cord. Intriguingly, recent studies suggest that caspases not only induce apoptosis but also promote neural development processes, including axon branching and synapse formation, independent of their apoptosis-related roles (D’Amelio et al., 2010; Mukherjee and Williams, 2017). However, the mechanisms underlying the choice by neural cells to activate either death-related or differentiation-promoting roles of caspases remain obscure.

In this review, we highlight the current knowledge on the apoptotic and non-apoptotic roles of caspases in the development of the CNS. We depict molecular aspects of caspase regulation, which might be critical for neural cell destiny during embryonic development and further discuss current “controversies” in the field.

## Overview of the Caspase Family of Apoptosis Executioners

In metazoans, apoptotic mechanisms are evolutionarily conserved and allow multicellular organisms to preserve tissue homeostasis (Ameisen, 2002). Ced-3 was the first apoptosis executioner discovered in *Caenorhabditis elegans* (Ellis and Horvitz, 1986). Its first mammalian homologs, interleukin-1β-converting enzyme in humans and nedd-2 in mice, were described shortly after (Yuan et al., 1993). Homologs of these proteins were also found in insects, namely Dcp-1 and Drice in Drosophila. These proteins were named caspases, which stands for Cysteine Aspartyl-Specific Proteinases, reflecting their specificity for sites containing aspartic acid peptide bonds, which they cleave through the cysteine present in their active site. So far, about 15 caspase family members have been described in humans and mice (Shalini et al., 2015). Of note, in mammals, certain caspases are implicated also in inflammation. In this respect, it has been found that a given caspase can contribute to either apoptosis or inflammation, but not both (Martinon and Tschopp, 2004; Shalini et al., 2015). This review focuses on apoptotic caspases and their roles in the development of the nervous system.

### Classification and Structure

**FIGURE 1:**
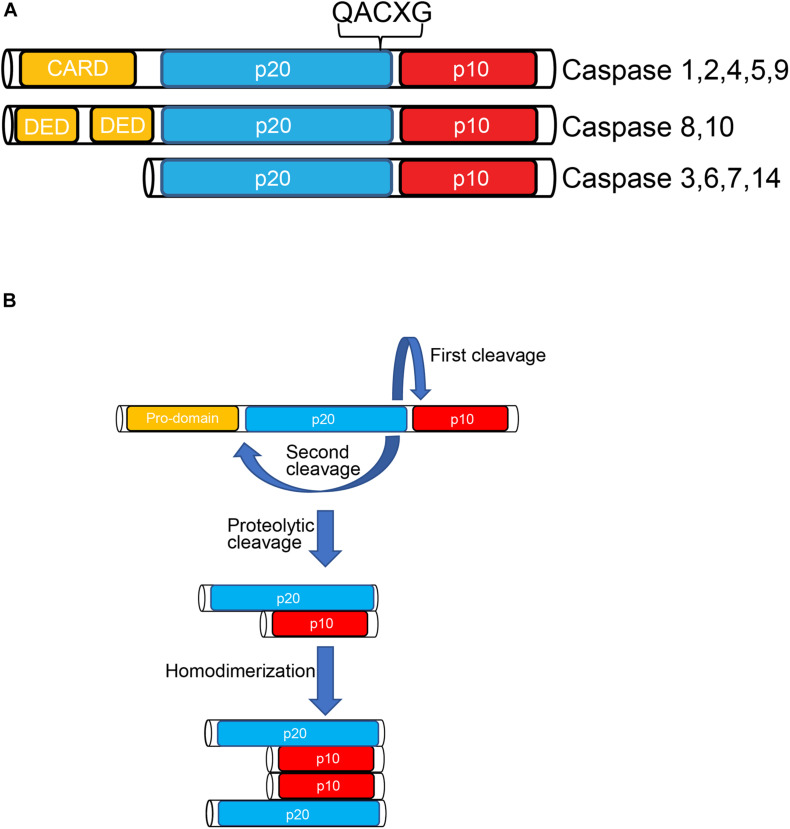
The caspase family of proteases. **(A)** Members of the caspase family, are synthesized as inactive precursors called zymogens composed of a prodomain, a p20 large subunit and a p10 small subunit. The p20 subunit contains the active site of the enzyme harboring a “QACXG” pentapeptide motif. Initiator Caspases are characterized by the presence of a long N-terminal prodomain whereas the N-terminus domain of effector caspases is shorter. **(B)** Caspase activation is achieved by proteolytic cleavage between the large and small subunits and removal of the N-terminus prodomain. This post translational modification leads to new conformational state in which caspase homodimers are fully active.

**FIGURE 2:**
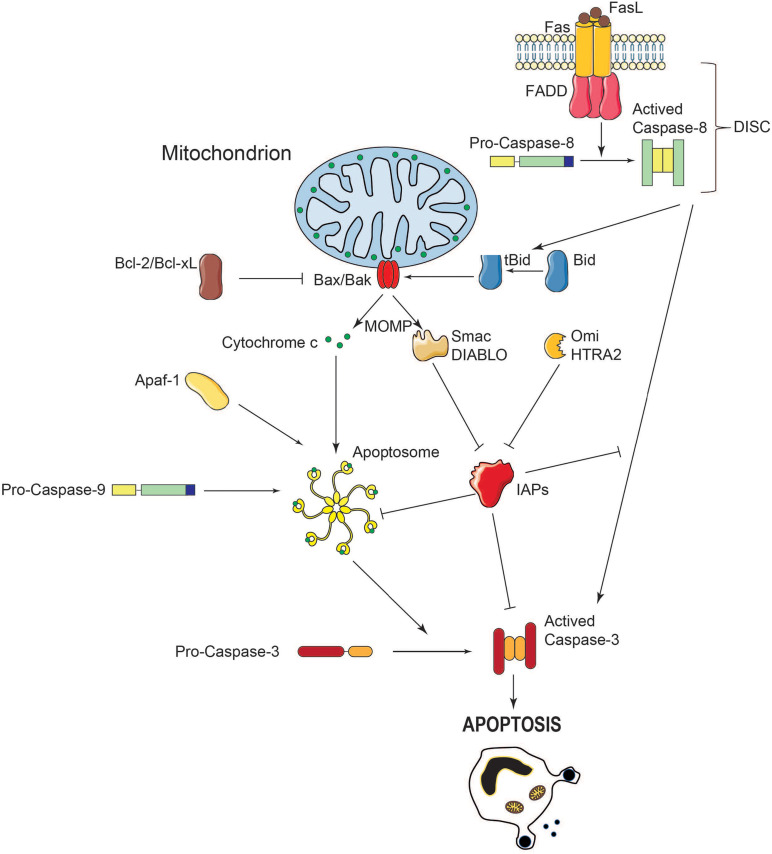
Schematic representation of caspase activation and regulation during MOMP. During apoptosis, the MOM is permeabilized, leading to the release of pro-apoptotic molecules into the cytosol. When released in the cytosol, cytochrome c interacts with the adaptor protein Apaf-1 in presence of ATP. A multiprotein complex called the apoptosome, comprising cytochrome c, Apaf-1 and caspase-9 activates caspase-3, leading to cell death. The IAPs prevent caspase activation and apoptosis whereas Smac/Diablo and HtrA2/Omi exert the opposite effect by reversible direct binding and/or proteolytic cleavage. Crosstalk between extrinsic and intrinsic apoptosis pathways takes place through caspase-8-mediated Bid cleavage which activates Bax and Bak and subsequent MOMP.

Apoptotic caspases, including those in humans, are classified into two groups: the initiators (caspase-8, -9, and -10) and the executioners (caspase-3, -6, and -7). These proteases are synthesized as proteolytically inactive zymogens. On the one hand, initiator caspases comprise an extended prodomain in the N-terminus moiety, upstream of the conserved region encoding the large and small subunits of the active site (Figure 1A). On the other hand, executor caspases have a short or even absent prodomain. During the process of initiator caspase activation, the prodomain and the linker region between both subunits, are removed, giving rise to a homodimer (namely, a tetramer) with two active sites (Figure 1B; Li and Yuan, 2008; Shalini et al., 2015). Initiator caspases can self-activate *via* autocatalysis, however, additional interactions with an activating protein platform is usually required. For instance, activation rate is several orders of magnitude higher when caspase-9 is docked in a supramolecular complex referred to as the apoptosome, compared to the free form (Rodriguez and Lazebnik, 1999). In this respect, two well-known activation platforms of initiator caspases are death-inducing signaling complex (DISC) and the apoptosome complex. The former activates caspase-8, and the latter activates caspase-9 (Figure 2). Unlike the initiators, executor caspases, lacking a proper prodomain, can dimerize shortly after being synthesized. However, the linker region between the large and small subunits prevents activation. Removal of the linker is performed by initiator caspases, such a caspase-8 and -9, after which activated executioners cleave a broad set of substrates, leading to apoptosis (Kumar, 2007).

### Caspase Substrates

Several hundreds of proteins are cleaved by caspases during apoptosis, but only a small proportion is considered to be of biological significance for cell remodeling (Julien and Wells, 2017). Among the most well-known targets of caspases are cytoskeletal proteins, including actin and tubulin (Mashima et al., 1999; Sokolowski et al., 2014), and nuclear envelope proteins, including lamins (Slee et al., 2001; Raz et al., 2006).

In fact, a number of caspase-substrates are involved in non-apoptotic processes, including cell proliferation and differentiation (Kuranaga and Miura, 2007). During such processes, cleavage of caspase targets remains strictly controlled in time and space, avoiding detrimental cell death. For instance, in erythropoiesis, the transcription factor GATA-1 is protected from caspase-mediated proteolysis by the chaperon protein Hsp70 (De Maria et al., 1999; Ribeil et al., 2007).

So far, an exhaustive view of the specific roles of each member of the caspase family is lacking. The precise map of their actual substrates also remains to be determined, although the preferred cleavage sites of each caspase were identified by proteomic studies. For instance, caspase-3 and -7 both cleave the same DEVD motif. However, caspase-3 was shown to be more efficient than caspase-7 in cleaving well-known substrates such as Bid, XIAP, and caspase-6. Moreover, the amplification of the apoptotic cascade through the caspase-9 feeding loop depends on caspase-3 but not on caspase-7 (Walsh et al., 2008).

## Mechanisms of Caspase Regulation

The caspase activation cascade occurs *via* two broadly recognized pathways, referred to as extrinsic and intrinsic. In the extrinsic pathway stimulation of death receptors such as Fas/Apo1 or TRAIL, leads to the activation of initiator caspase-8 and downstream cleavage of effector caspase-3. The intrinsic pathway involves the mitochondria, and notably the release of cytochrome c from the mitochondrial intermembrane space into the cytosol. Released cytochrome c binds to the Apaf-1 docking protein and facilitates the formation of the apoptosome complex that recruits and activates caspase-9. This caspase-9/Apaf-1/cytochrome c apoptosome complex is the holoenzyme form of caspase-9, which activates the caspase-3 apoptosis executioner by proteolytic cleavage (Figure 2; Hengartner, 2000).

### Bcl-2 Family Proteins

Mitochondria play a central role in caspases activation through the intrinsic pathway (Singh et al., 2019). Upon various death-inducing stimuli the mitochondrial outer membrane (MOM) is altered, allowing cytochrome c release into cytosol and subsequent activation of the caspase cascade. This phenomenon, referred to as mitochondrial outer membrane permeabilization (MOMP), is strictly regulated by the Bcl-2 protein family (Kale et al., 2018).

Bcl-2 proteins share one or multiple conserved Bcl-2 homology (BH) domains (Youle and Strasser, 2008). Based on structure and function analyses, Bcl-2 proteins have been classified into three groups: BH3-only, pro-apoptotic multidomain, and anti-apoptotic multidomain (Youle and Strasser, 2008; Chipuk et al., 2010). Once activated, pro-apoptotic multidomain proteins, such as Bax and Bak, form homo- and hetero-oligomeric channels on the MOM, inducing the leakage of apoptogenic agents into the cytosol (Bleicken et al., 2013; Zhang et al., 2017). Anti-apoptotic multidomain Bcl-2 homologs, including Bcl-2, Bcl-xL, and Mcl-1, neutralize Bax and Bak through a hydrophobic groove formed by their BH1, BH2 and BH3 domains, therefore protecting the cell from apoptosis (Petros et al., 2004). BH3-only proteins are pro-apoptotic proteins that play the role of “sentinels” capable of integrating diverse cellular stresses and of shifting the balance between pro- and anti-apoptotic toward MOMP by antagonizing anti-apoptotic Bcl-2 family members or activating Bax and Bak (Youle and Strasser, 2008).

### Caspase Inhibitors

Caspase activity is also controlled by the inhibitor of apoptosis proteins (IAPs). Such control may occur *via* either direct inhibition or degradation through ubiquitination and downstream ubiquitin-targeted proteasome machinery (Joazeiro and Weissman, 2000; Zhang et al., 2019). Intriguingly, X-linked IAP (XIAP) was also shown to be a direct caspase inhibitor, independent of ubiquitin. Indeed, XIAP inhibits the enzymatic activity of both initiator and executor caspases (Eckelman et al., 2006). Following MOMP-induction, other apoptogenic molecules aside from cytochrome c, including Smac/DIABLO, HtrA2/Omi, AIF, and endonuclease G are also released into the cytosol. In turn, Smac/DIABLO and HtrA2/Omi neutralize the IAPs, which results in caspase-induced cell remodeling (Du et al., 2000).

### Regulation Through Phosphorylation

Both initiator (capase-8 and -9) and executioner caspases (caspase-3 and -7) are directly regulated by phosphorylation (Parrish et al., 2013; Zamaraev et al., 2017). Caspase-3 activity has been reported to be inhibited by p38MAPK though phosphorylation of the highly conserved S150 residue, which is reversed by protein phosphatase 2A (PP2A) (Alvarado-Kristensson et al., 2004; Alvarado-Kristensson and Andersson, 2005). Caspase-9 has the largest subset of reported phosphorylation sites. Most of these sites are located in the large and small subunits but not in the active site (Zamaraev et al., 2017). Tyr153 is the only phosphorylation site reported to promote caspase-9 activation whereas other sites impede auto-processing and block substrate binding (Raina et al., 2005; Allan and Clarke, 2007; Serrano et al., 2017; Serrano and Hardy, 2018). Both inhibitory and activating phosphorylation sites are present in caspase-3, whereas only the former have been described in caspase-7 (Alvarado-Kristensson et al., 2004; Voss et al., 2005; Eron et al., 2017). However, the regulation of caspase activity by phosphorylation remains to be formally demonstrated in nerve cells.

## Apoptotic Role of Caspases in the Developing Nervous System

### Control of Neural Tube Closure

Numerous apoptotic events occur during the development of the nervous system in vertebrates and invertebrates. At an early developmental stage, apoptotic cells are found at the boundaries between the non-neural ectoderm and the neuroepithelial layer during and after neural tube closure (NTC) (Geelen and Langman, 1977; Weil et al., 1997). Interestingly, apoptosis takes place throughout the major steps of NTC, including neural fold bending and fusion processes, remodeling of the dorsal neural tube and of the ectoderm, as well as migration of neural crest cells away from the neural tube (Massa et al., 2009; Washausen et al., 2018). Indeed, suppression of apoptosis with caspase inhibitors entirely impedes the NTC in the chick embryo (Weil et al., 1997). Furthermore, there is evidence of a potential link between mutations in apoptosis-related genes (*caspase-3, caspase-9*, and *apaf-1*) and neural tube closure defects in human (Liu et al., 2018; Spellicy et al., 2018; Zhou et al., 2018).

In mice, *caspase-3*- and *apaf-1*-null embryos only show neural tube closure defects in the midbrain and the hindbrain, while neurulation proceeds normally in the forebrain and the spinal cord. Interestingly, pharmacological inhibition of apoptosis using the pan caspase inhibitor z-VAD-FMK or the p53 inhibitor Pifithrin-α does not impact neural tube closure in *ex vivo* cultured embryos (Massa et al., 2009). This observation suggests that the observed neural tube closure defect may not be a direct consequence of the suppression of apoptosis but could be due to the abnormal persistence of certain signal-secreting cell populations that should have been eliminated by apoptosis. Indeed, this may explain why neural tube malformations were not observed in *ex vivo* cultures, where secreted factors are expected to be rapidly diluted in the culture medium.

### Control of Cell Population Size

Apoptosis is widely recognized as main regulator of cell number during development. In developing CNS, neurons are generated excessively. Then, this population is trimmed down *via* apoptosis: neurons without proper connection with their target are eliminated. In the context where apoptosis is suppressed or compromised, the nervous system, especially the brain, would be overloaded with neurons, leading to protrusions and ventricular obstruction (Jacobson et al., 1997; Buss et al., 2006).

Several knock out mouse models demonstrated the critical role of caspase-dependent apoptosis in controlling the size of neuron populations during development. *Caspase-3* invalidation in 129 × 1/SvJ mice leads to exencephaly, a defect in which the brain exhibits abnormal protrusions and ventricular absence due to hyperplasia (Kuida et al., 1996). Moreover, *caspase-9*-null embryos showed prenatal malformations in the brain and the spinal cord, similar to those observed in *caspase-3*-null embryos, including protrusion formation and ventricular obstructions. Both *caspase-3*- and *caspase-9*-null mice die rapidly after birth (Kuida et al., 1998). Of note, these mice show an abnormal increase in neuron number, suggesting that MOMP is indeed required for neuronal death during the development of the CNS. Interestingly, invalidation of *apaf-1*, a gene coding for a key component of the apoptosome, results in more severe hyperplasia phenotype than in *caspase-3*- or *caspase-9*-null mice, further supporting the role of the intrinsic apoptosis pathway in brain homeostasis (Cecconi et al., 1998; Yoshida et al., 1998). Finally, mouse embryonic fibroblasts derived from *cytochrome c*-null embryos failed to activate caspase-3 in response to different apoptotic stimuli. Of note, the latter embryos exhibited delayed development, dying at E10.5 (Li et al., 2000). However, it remains unclear if these effects are due to apoptosis failure or mitochondrial electron transport chain deficiency.

Regarding the extrinsic pathway of apoptosis, even though *caspase-8* invalidation, causes heart malformation erythrocyte congestion, and neural tube defects, leading to embryonic death at E13.5, the malformations observed in the nervous system are conceivably not directly linked to *caspase-8* silencing (Varfolomeev et al., 1998; Sakamaki et al., 2002).

It should also be noted that malformations of the neural tube induced by the invalidation of *caspase-3* and *apaf-1* are mouse-strain specific. Indeed, contrary to 129 × 1/SvJ mice, *caspase-3*-null C57BL/6J mice showed no macroscopic anomalies and reached adulthood without any detectable brain pathology (Leonard et al., 2002). Similar strain-dependent effect was also observed in *apaf-1*-null mice. In effect, C57BL/6J and CD1 mice show significantly delayed lethality compared to 129/Sv mice. These controversial observations might be due to some redundancy between effector caspases. Actually, although neither caspase-6 nor caspase-7 appear to be critical for CNS development, caspase-7 is capable to trigger apoptosis in a caspase-3-independent manner and is potentially responsible for the “rescued” phenotype in C57BL/6J *caspase-3*-null mice (Houde et al., 2004; Lakhani, 2006; Uribe et al., 2012).

Finally, although this issue was not addressed in above studies, compensation by other types of PCD such as autophagy and necroptosis cannot be eliminated.

The notion that, during development, cell number is exclusively controlled by apoptosis has recently been challenged by Nonomura et al. (2013). In this study, apoptosis suppression by *caspase-9* or *apaf-1* invalidation, even in a 129S1 background, did not lead to an increase of nerve cell number, whereas severe brain malformations were still observed. Actually, the size of neuron populations would be controlled by proliferation rate and other types of PCD.

In contrast, apoptosis was shown to be essential to control the FGF-8-producing cell population. Indeed, after the NTC, apoptosis is maintained in the midline of the neural tube, where these cells are localized. During development, activation of apoptosis around day E10 leads to the removal of these FGF-8-producing cells, preventing ectopic diffusion of FGF-8 to the ventral telencephalon (Nonomura et al., 2013). Together, these observations support the notion that the roles of apoptosis in the developing nervous system are actually cell-type specific.

## Non-Apoptotic Roles of Caspases in the Developing Nervous System

### Axon Branching and Arborization

Recent studies proposed that caspases, particularly caspase-3, play multiple roles aside from apoptosis. Activation of caspase-3 has been observed transiently and locally in the axons, notably at the level of branching points (Campbell and Okamoto, 2013; Katow et al., 2017). Intriguingly, this transient activation of caspase-3 did not always lead to apoptosis of the host neuron. Inhibition of caspase-3 in these models resulted in a decrease in the number of axonal branches. Caspase-3 cleavage was also shown to mediate the growth cone formation (Campbell and Holt, 2003; Verma, 2005).

The role of caspase-3 in axon regeneration was further highlighted in a model of axon disruption in *Caenorhabditis elegans*. In the latter, the homolog of vertebrate caspase-3 is encoded by the *ced-3* gene. Using neurons from a *ced-3*-null *C. elegans* strain, Pinan-Lucarre et al. (2012) showed that axons had lower outgrowth rate, being unable to re-connect after axotomy. Recently, in *C. elegans* Wang et al. (2019) successfully detected increased ced-3 activity following axotomy which was correlated to axon regeneration. Moreover, in neurons derived from rat DRG, caspase-3 inhibitors were found to impact growth cone formation, which is critical for restoring injured axons (Verma, 2005).

The mechanisms by which caspase-3 regulates growth cone formation and axon branching are still elusive. However, the contribution of the cytoskeleton is presumably important. In axons and dendrites, cytoskeleton components comprise actin microfilaments, microtubules, and intermediate filaments. In developing neurons, actin filaments are mainly found in growth cones. In contrast, microtubules are evenly distributed along the axons. However, it should be noted that the rate of microtubule remodeling is much higher in the axon terminus, compared to axon shafts. The growth cone is a guiding structure at the terminus of developing axons, characterized by actin enriched structures referred to as filopodia and lamellipodia (Pacheco and Gallo, 2016). Most interestingly, cytoskeletal actin was reported to be a substrate for caspase-3. Actin cleavage by caspase-3 gives rise to a 15 kD fragment inducing a more condensed and fragmented actin network (Mashima et al., 1999). Moreover, caspase-3 was demonstrated to cleave Rho-associated kinase ROCK I, generating a truncated form with higher intrinsic kinase activity. Such activated ROCK I stabilizes actin microfilaments, phosphorylates myosin light chains, and promotes the coupling between actin and myosin filaments, causing cellular contractions (Coleman et al., 2001). Thus, in the context of neuronal cells, caspase-3 may exert the same function in order to stabilize the cytoskeleton of new axon branches.

Aside from actin microfilaments, intermediate filaments are also potential targets of caspase-3 in axons. Immature neurons mainly express vimentin and nestin as intermediate filaments, which are replaced by neurofilaments in mature neurons. Beside their role as scaffolding structures, vimentin and nestin function as adaptors, contributing to axon growth cues (Adolf et al., 2016; Bott et al., 2019).

In addition to the cytoskeleton, caspases may further contribute to axon guidance through their impact on cell adhesion molecules, such as Neural cell adhesion molecule (NCAM), and extracellular vesicle proteins (Westphal et al., 2010; Weghorst et al., 2020).

### Axon Pruning

Axon pruning is a process that eliminates collateral extensions or small terminus arborization with improper connectivity at the axon terminus. This process occurs at embryonic or early postnatal stage to fine-tune neural connectivity and has been well described in the peripheral nervous system (PNS). Axon pruning can be reproduced *in vitro* through the withdrawal of trophic factors such as nerve growth factor (NGF) or brain-derived neurotrophic factor (BDNF) from neuron cultures, including from DRG. In the developing CNS, although still poorly understood, similar mechanisms of network fine-tuning may occur at early postnatal stage (Innocenti and Price, 2005; De León Reyes et al., 2019).

In the sympathetic and DRG models, caspase-6 is widely recognized as an active contributor to axon pruning (Nikolaev et al., 2009; Cusack et al., 2013; Unsain et al., 2013). In contrast, the role of caspase-3 was initially overlooked. In the study of Nikolaev et al. (2009), activation of caspase-3 was not detected in axons using an immunofluorescent marker or the fluorescent reporter FAM-DEVD-fmk in sympathetic neurons in culture, following NGF withdrawal. Furthermore, inactivation of caspase-3 with pharmacological inhibitors or siRNAs did not prevent caspase-6 cleavage and axon degeneration.

However, recent reports from different laboratories highlighted the implication of caspase-3 in axon pruning, using genetic approaches. These latter studies demonstrated that *caspase-3* KO prevented axon degeneration in NGF-deprived neurons. Interestingly, *in vitro* studies showed that, caspase-6 can be only processed by caspase-3, even at low levels of active caspase-3 (Simon et al., 2012; Cusack et al., 2013; Unsain et al., 2013). The difference between these observations and the ones of Nikolaev might be due to the small amounts of activated caspase-3 in this context, which cannot be detected by immunofluorescence. In fact, small remaining amounts of active caspase-3, having escaped pharmacological inhibitors or siRNA are presumably sufficient to promote the observed caspase-6-dependent pruning.

The roles of caspases in axonal network fine-tuning remains to be further investigated. Recently, regarding *in vivo* studies, caspase-3 has been shown to be activated in the axon and not in the cell body of spinal cord neurons of postnatal mice. Suppression of caspase-3 activation by *bax/bak* double KO led to a less tailored network of neuron branches in the spinal cord at postnatal day 14, which disturbed the reorganization of the corticospinal circuit and consequently impaired the development of skilled movements in adult mice (Gu et al., 2017).

### Synapse Maturation and Synaptic Functions

Recent studies have suggested that, aside from refining the developing axonal network, caspases also promote fine-tuning of protein structure at the level of the synapses, which may be critical for their functioning. Synapses are at the key player structures regarding neuron communication. There are two existing types of synapses referred to as electrical and chemical, the latter being the most abundant in the CNS. In electrical synapses, signals are transmitted by an electrical current through gap junctions. In chemical synapses, communication between two neurons is performed by the secretion of a neurotransmitter in the synaptic cleft. Once released into the synaptic cleft, the neurotransmitter binds to receptors at the post-synaptic level and induces electrical signals by acting on ion channel permeability (Purves et al., 2018).

In the PNS, caspase-3 fine-tunes the post-synaptic neurotransmitter receptor network. During development, this process namely occurs at the neuromuscular junction, regarding the nicotinic acetyl choline receptor (AchR). Indeed, at early stages of development, AchRs are abundantly distributed. Later on, AchRs gather into clusters that are stabilized by agrin released from axon terminals. Moreover, motor neurons secrete acetylcholine, which acts as a negative signal, disrupting AChR clusters that do not interact with axon terminals. Of note, this disrupting effect of acetylcholine is mediated by caspase-3 which cleaves Disheveled 1 (Dvl1), a signaling protein of the Wnt pathway. Indeed, prevention of caspase-3 activation or Dvl1 cleavage suppresses acetylcholine-dependent AChR cluster disruption (Wang et al., 2014).

Certain similarities between synaptogenesis processes in the CNS and the PNS have been described, such as clustering of neurotransmitter receptors at the post-synaptic level (Garner et al., 2002; Purves et al., 2018). Regarding the underlying molecular mechanisms, agrin-like molecules and Ephrin-Bs have been reported to contribute to the clustering of *N*-methyl-D-aspartate (NMDA) and α-amino-3-hydroxy-5-methyl-4-isoxazolepropionic acid (AMPA) receptors, respectively (Garner et al., 2002). It remains to be explored if the role of caspase-3 in sculpturing post-synaptic receptor clusters in the PNS is conserved in the CNS.

Caspase-3 is also involved in synaptic plasticity, notably in long-term depression (LTD), a process during which the efficiency of synaptic transmission is lowered in the long-term. This process was thoroughly studied in the context of the glutamatergic synapse. The post-synaptic region of these synapses contains AMPA and NMDA receptors, which both respond to glutamate. Coordination between both receptors is critical for optimal synaptic transmission. In LTD, the AMPA receptor is withdrawn from the post-synaptic membrane by endocytosis, which leads to a decrease in synaptic sensitivity (Purves et al., 2018). Interestingly, caspase-3 activation is essential for AMPA receptor internalization at the synapse in response to NMDA stimulation *in vitro* (Li et al., 2010). Inhibition of the XIAP caspase-3 inhibitor enhances AMPA receptor internalization and increases LTD (Gibon et al., 2016). In addition, caspase-3 mediates synapse loss upon long-term exposure to NMDA (Henson et al., 2017). Furthermore, local activation of caspase-3 induced by Mito-killer Red photo-stimulation results in local spine shrinkage and subsequent elimination of spines without neuronal apoptosis (Erturk et al., 2014). Dendritic spine regulation by BDNF is also mediated by caspase-3 (Guo et al., 2016). *In vivo*, *caspase-3* KO mice display a lack of attention and hyperactive disorder, potentially related to a failure in synaptic plasticity mechanisms, such as AMPA receptor regulation in response to chronic or repeated stimuli (Lo et al., 2015). In contrast, *xiap* KO mice exhibit better learning performance, which could be explained by increased LTD (Gibon et al., 2016).

In addition, caspase-3 regulates synaptic vesicle pool and eliminates dysfunctional dendritic spines (Chen et al., 2020). Collectively, these data highlight the central roles of caspase-3 in synaptic plasticity and functions.

### Local and Temporal Activation of Caspases in Non-apoptotic Processes

Activation of caspases in the above described non-apoptotic contexts seem to be both local and transient. Most of the time, ultimate caspase-3 activation is required for axon remodeling and synapse functions. Mitochondria present at axon terminals were reported to be critical for axon branching (Courchet et al., 2013), suggesting that such caspase-3 activation presumably depends on the mitochondrial pathway. Indeed, the release of cytochrome c into the cytosol appears to occur prior to local caspase-3 activation (Li et al., 2010; Guo et al., 2016). Finally, caspase-9 inhibition was shown to prevent such local caspase-3 activation, further confirming the contribution of mitochondria (Ohsawa et al., 2010; Erturk et al., 2014; Khatri et al., 2018). Intriguingly, even though the role of caspase-9 in local caspase-3 processing appears to be clearly established in this context, the role of the apoptosome remains unclear (Ohsawa et al., 2010; Cusack et al., 2013).

**FIGURE 3:**
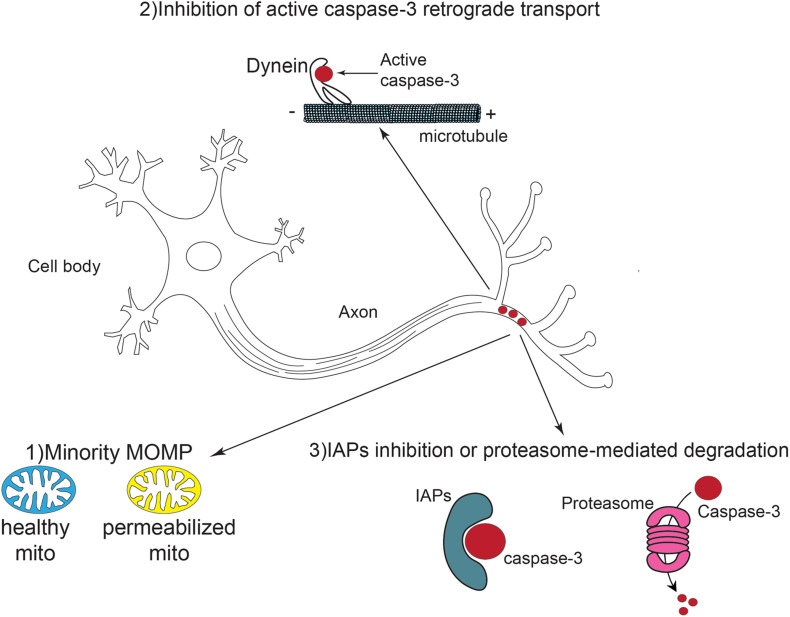
Schematic representation of local caspase activation and potential regulation pathways in neural development. Caspase-3 is activated locally and transiently in the axons of developing and mature neurons. This activation might result from a phenomenon called “minority MOMP” in which only a subpopulation of mitochondria is permeabilized while the others remain healthy. The retrograde transport of active caspases *via* dynein is also inhibited. Finally, IAPs can suppress caspase-3 activity through direct inhibition or proteasome mediated degradation.

Although most studies acknowledge that the mitochondrial pathway is the key player in caspase-3 activation in non-apoptotic contexts, it remains unclear how neurons can control caspase-3 activity locally and temporally. *A priori*, several scenarios are possible (Figure 3).

First, it is likely that a limited number of mitochondria are actually permeabilized and release cytochrome c, to allow such sub-optimal caspase activation. MOMP was widely thought to be a point of no-return from which cells commit suicide. There is evidence that apoptotic signals can be initiated by a limited number of mitochondria and then spread throughout the cell in “apoptotic waves” (Pacher and Hajnóczky, 2001; Cheng and Ferrell, 2018). In axons, mitochondria are more distant compared to the cell body. This topological barrier might result in the permeabilization of a fraction of the mitochondria, leaving most of them intact and healthy.

The mechanisms underlying the propagation of “apoptotic waves” is a matter of debate. Indeed, Ca^2+^ may play a role in this respect. Aside from cytochrome c and cognate apoptogenic agents, depolarized mitochondria initiate Ca^2+^ waves and trigger the depolarization of surrounding mitochondria (Pacher and Hajnóczky, 2001; Ziegler et al., 2021). Actually, the propagation of waves of released cytochrome c have been reported during apoptosis (Lartigue et al., 2008; Cheng and Ferrell, 2018). Such a propagation of waves of cytochrome c into the cytosol seems to independent of pro-apoptotic proteins Bax and Bak. Intriguingly, the presence of cytochrome c in the cytosol failed to trigger cell death in sympathetic neurons (Deshmukh and Johnson, 1998). Overall, these observations suggest that additional mechanisms controlling apoptosis are presumably involved in neuronal cells. Indeed, in post-mitotic neurons, an E3 ligase, referred to as Cullin 9 (CUL9) or Parkin-like cytoplasmic protein (PARC) was reported to mediate cytosolic cytochrome c degradation (Gama et al., 2014), suggesting that, in neurons, the propagation of cytosolic cytochrome c may be blocked by proteasome-mediated degradation.

Of note, partial cytochrome c release has been described in physiological contexts, beyond apoptosis. Basically, under certain conditions, MOMP may occur only in some mitochondria while the others remain intact (Tait et al., 2010). Incomplete MOMP appears to have physiological effects in some instances. For example, microbial infection stimulates MOMP in a fraction of mitochondria of the host cell, allowing sublethal caspase activation to promote cytokine secretion in the context of an innate immune response (Brokatzky et al., 2019). Thus, partial caspase activation might occur through similar mechanisms in developing neurons (Figure 3).

Second, in neurons, it can be anticipated that some mechanisms may have been set up to avoid uncontrolled propagation of active caspases. Indeed, there are shreds of evidence of cleaved caspase retrograde transport *via* the microtubule network (Carson et al., 2005; García et al., 2013; Barreiro-Iglesias et al., 2017). Activated caspase-8 was demonstrated to interact with the dynactin/dynein complex, which is responsible for retrograde axonal transport, and to be transferred in this way from axon terminals to the cell body (Carson et al., 2005; Barreiro-Iglesias et al., 2017). Thus, retrograde movement of caspase-8 leads to caspase-3 cleavage along the axons as well as in the cell body. Activated caspase-3 was also found to directly associate with dynein, suggesting that it can be transported back to the soma through a similar mechanism (García et al., 2013). In non-apoptotic contexts, including neuron differentiation, the interaction between activated caspase-3 and the dynactin/dynein complex might thus be inhibited.

Third, active caspases, after having performed their extra-apoptotic mission, must be rapidly neutralized to avoid deleterious alterations of cell components due to uncontrolled proteolysis. The IAP family of caspase inhibitors, including XIAP, may contribute to such neutralization. Indeed, in dorsal root ganglio (DRG) neurons, XIAP is localized in axons and slackens off caspase-3-dependent axonal degeneration (Unsain et al., 2013). Caspases can also be eliminated through IAP-mediated ubiquitinylation and proteasomal degradation. Evidence indicates a dynamic equilibrium of proteasome anterograde and retrograde transport in neurons. Proteasome components are actively transported to distal axons *via* kinesin motors (Otero et al., 2014). Indeed, the presence of the proteasome at the end of axons is essential to maintain protein homeostasis. This is highlighted in certain neurodegenerative pathologies such as Alzheimer’s disease, in which proteasomal degradation is aberrant along the axons. On the contrary, in nascent synapses, proteasome components are retrogradely transported *via* dynein motors, which permits the stabilization of synaptic structures (Hsu et al., 2015). Apparently, by controlling anterograde and retrograde transports, the guidance of proteasome components in axons is precisely controlled in accordance with intra- and extra-cellular cues. Thus, it might be assumed that local proteasome recruitment may occur to regulate caspase activation during axonal growth or branching.

## Bcl-2 Family: Potential Regulators of Caspase Roles in the Developing CNS

### Apoptosis and Mitochondria

As mentioned above, it has long been acknowledged that the Bcl-2 family regulates caspase activation *via* the mitochondrial pathway of apoptosis. Pore formation in the MOM by pro-apoptotic proteins Bax and Bak is a key step of this canonical pathway. These pores permit the release of cytochrome c and other apoptogenic molecules into the cytosol. In addition, Bax and Bak also mediate the release of Ca^2^**^+^** from the endoplasmic reticulum (ER) lumen (Scorrano et al., 2003). Consequently, mitochondria are overloaded with Ca^2^**^+^**, which further induces the mitochondrial permeability transition pore (MPTP) (Giorgi et al., 2012). The nature of MPTP is still ambiguous, however, it seems to act synergistically with the Bax/Bak pores to foster the release of intermembrane-space components into the cytosol (Zhang and Armstrong, 2007). Finally, it is noteworthy that the anti-apoptotic proteins, including Bcl-2, Bcl-xL, Mcl-1, and Bcl-w, antagonize Bax and Bak in both mitochondria- and Ca^2^**^+^**-dependent PCD pathways.

In the developing CNS, Bax, Bak, Bcl-xL, and Mcl-1 are expressed at a high level (Lindsten et al., 2000; Arbour et al., 2008; Nakamura et al., 2016). Bcl-2 is also found to be highly expressed during embryonic development, although *bcl-2* invalidation does not seem to affect the development of the CNS in mice (Michaelidis et al., 1996). Bax and Bak apoptosis accelerators often show overlapping roles. *Bax* invalidation does not entirely eliminate apoptosis in the developing CNS but affects some specific neural populations, including neuroblasts, retinal ganglion cells and Cajal–Retzius neurons (Knudson et al., 1995; Péquignot et al., 2003; Ledonne et al., 2016). *Bak* KO mice develop normally without detectable malformation in the CNS (Lindsten et al., 2000). *Bax/bak* double KO leads to perinatal lethality in about 90% homozygous offspring (Lindsten et al., 2000).

There is evidence that the loss of Bax and Bak, can be partly compensated by the apoptosis accelerator Bok, a multidomain Bcl-2 homolog whose functions remain poorly understood (Ke et al., 2018).

Although ventricular obstruction was observed due to an abnormal increase of neuron number in *bax/bak* double KO, these mice did not exhibit exencephaly, unlike *caspase-3*- or *caspase-9*- null mice. Interestingly, interdigital webs and imperforate vagina were observed in *bax/bak* double KO, but not in *caspase-3*- or *caspase-9*- null mice (Lindsten et al., 2000). These diverging phenotypes presumably underscore the role of Bax and Bak roles in the other types of PCD (Kawai et al., 2009; Karch et al., 2013, 2017).

Conversely, regarding cell death inhibitors, the two anti-apoptotic Bcl-2 homologs Mcl-1, and Bcl-xL, appear to hamper apoptotic cell death in neurons. Indeed, Mcl-1 is critical for the survival of neural progenitors (Arbour et al., 2008), whereas Bcl-xL protects post-mitotic neurons from cell death (Nakamura et al., 2016). Of note, the full KO of *bcl-x* leads to embryonic lethality due to massive apoptosis in the CNS (Motoyama et al., 1995; Fogarty et al., 2016; Nakamura et al., 2016).

### Incomplete MOMP and Non-apoptotic Functions

Bcl-2 proteins have also been found to regulate incomplete MOMP in several instances (Singh et al., 2019; Bock and Tait, 2020). A suggested model for incomplete MOMP is based on the heterogeneous distribution of Bcl-2 proteins in the MOM. According to this model, mitochondria harboring high amounts of anti-apoptotic proteins, such as Bcl-2 and Bcl-xL, would be protected from Bax and Bak-induced permeabilization, whereas mitochondria deprived of apoptosis inhibitors would be privileged prey for Bax and Bak (Tait et al., 2010).

The mechanism allowing such heterogeneity is still unclear. It might involve a signal to recruit Bax/Bak to a specific population of mitochondria and/or a signal to shift anti-apoptotic proteins either to another mitochondrial pool or to the cytosol. In line with this, the retromer is a system conceivably able to mediate the translocation of anti-apoptotic proteins to distinct mitochondrial populations. Indeed, a recent study showed that Bcl-xL forms a complex with VPS35/VPS26 retromer proteins. This complex promotes the mitochondrial localization of Bcl-xL (Farmer et al., 2019). Thus, a signal may be present on mitochondria to recruit this Bcl-xL-retromer complex. Anti-apoptotic proteins might also be eliminated from mitochondria through Mitochondrial-anchored protein ligase (MAPL)-mediated mitochondria-derived vesicles, a structure transferring MOM proteins from the mitochondria to the peroxisome (Neuspiel et al., 2008).

In mature neurons, moderate Bax activation is observed in response to NMDA receptor-dependent LTD. Interestingly, in this instance, translocation of Bax to mitochondria could not be detected, suggesting that only a minor fraction of Bax, already present at the level of the mitochondria, was activated. Alternatively, Bax translocation might be too low to be detected with current methods (Jiao and Li, 2011). Nevertheless, such partial Bax activation may also occur in developing neurons.

### Mitochondrial Dynamics

Finally, Bcl-2 proteins were reported to control mitochondrial morphology and motility. Indeed, Bcl-xL increases both the fusion and fission rate of mitochondria (Berman et al., 2009). Furthermore, Bcl-xL interacts with dynamin-related protein (Drp1) and promotes the transport of mitochondria in axons (Li et al., 2008). In hippocampal neurons, this interaction favors mitochondrial fission to form “tiny mitochondria” capable of being distributed to new axon branches. It was also reported that inhibition of Bcl-xL by ABT-737 lowers the number of mitochondria along the axon, decreasing synapse number and synaptic vesical clusters.

## Conclusion

Caspase-mediated apoptosis has been considered as an essential mechanism regulating CNS development in vertebrates for years. Recent studies propose new insights into how apoptosis shapes the mammalian nervous system. These new observations challenge the conventional model of cell number control. Current data suggest that apoptosis may affect the behavior of neighboring cells or trim down a specific cell population (Nonomura et al., 2013; Yamaguchi and Miura, 2015). Genetically-modified mouse models have highlighted the role of caspase-3 as the main caspase effector in the developing CNS. However, why the effect of *caspase-3* invalidation in mice is strain-specific is still an open question. This is possibly due to the compensation of other effector caspases, such as caspase-7 (Houde et al., 2004). In this respect, compensation mechanisms either from other types of PCD, such as autophagy, or proliferation inhibition by senescence, should also be considered.

Novel techniques allowing to detect slight and transient caspase-3 activation in neurons have been recently documented. It is conceivable that such activation is physiologically relevant in the context of neurogenesis and day-to-day synaptic functions. The existence of two “opposite” effects of caspase-3 activation suggests that nerve cells may be equipped with the adequate regulation pathways to engage in distinct cell fates. The Bcl-2 family protein is a potential candidate to regulate these pathways. As Bcl-2 proteins control caspase-3 activation through MOM permeability, they may also allow incomplete MOMP and subsequent caspase-3 activation at low levels. The upstream mechanisms underlying this Bcl-2 family-dependent incomplete MOMP are currently unknown. They definitely deserve to be further studied in the near future.

## Author Contributions

TN, GG, and NP wrote the manuscript. All authors contributed to the article and approved the submitted version.

## Conflict of Interest

The authors declare that the research was conducted in the absence of any commercial or financial relationships that could be construed as a potential conflict of interest.

## References

[B1] Adolf A. Leondaritis G. Rohrbeck A. Eickholt B. J. Just I. Ahnert-Hilger G. (2016). The intermediate filament protein vimentin is essential for axonotrophic effects of *Clostridium botulinum* C3 exoenzyme. *J. Neurochem.* 139 234–244. 10.1111/jnc.13739 27419376

[B2] Allan L. A. Clarke P. R. (2007). Phosphorylation of caspase-9 by CDK1/cyclin B1 protects mitotic cells against apoptosis. *Mol. Cell* 26 301–310. 10.1016/j.molcel.2007.03.019 17466630

[B3] Alvarado-Kristensson M. Andersson T. (2005). Protein phosphatase 2A regulates apoptosis in neutrophils by dephosphorylating both p38 MAPK and its substrate caspase 3^∗^. *J. Biol. Chem.* 280 6238–6244. 10.1074/jbc.M409718200 15569672

[B4] Alvarado-Kristensson M. Melander F. Leandersson K. Rönnstrand L. Wernstedt C. Andersson T. (2004). p38-MAPK signals survival by phosphorylation of caspase-8 and caspase-3 in human neutrophils. *J. Exp. Med.* 199 449–458. 10.1084/jem.20031771 14970175 PMC2211830

[B5] Ameisen J. C. (2002). On the origin, evolution, and nature of programmed cell death: a timeline of four billion years. *Cell Death Differ.* 9 367–393. 10.1038/sj.cdd.4400950 11965491

[B6] Arbour N. Vanderluit J. L. Le Grand J. N. Jahani-Asl A. Ruzhynsky V. A. Cheung E. C. C. (2008). Mcl-1 is a key regulator of apoptosis during CNS development and after DNA damage. *J. Neurosci.* 28 6068–6078. 10.1523/JNEUROSCI.4940-07.2008 18550749 PMC2681190

[B7] Ayala R. Shu T. Tsai L.-H. (2007). Trekking across the brain: the journey of neuronal migration. *Cell* 128 29–43. 10.1016/j.cell.2006.12.021 17218253

[B8] Barreiro-Iglesias A. Sobrido-Cameán D. Shifman M. I. (2017). Retrograde activation of the extrinsic apoptotic pathway in spinal-projecting neurons after a complete spinal cord injury in lampreys. *BioMed. Res. Int.* 2017:5953674. 10.1155/2017/5953674 29333445 PMC5733621

[B9] Berman S. B. Chen Y. Qi B. McCaffery J. M. Rucker E. B. Goebbels S. (2009). Bcl-xL increases mitochondrial fission, fusion, and biomass in neurons. *J. Cell Biol.* 184 707–719. 10.1083/jcb.200809060 19255249 PMC2686401

[B10] Bleicken S. Landeta O. Landajuela A. Basañez G. García-Sáez A. J. (2013). Proapoptotic bax and bak proteins form stable protein-permeable pores of tunable size. *J. Biol. Chem.* 288 33241–33252. 10.1074/jbc.M113.512087 24100034 PMC3829170

[B11] Bock F. J. Tait S. W. G. (2020). Mitochondria as multifaceted regulators of cell death. *Nat. Rev. Mol. Cell Biol.* 21 85–100. 10.1038/s41580-019-0173-8 31636403

[B12] Bott C. J. Johnson C. G. Yap C. C. Dwyer N. D. Litwa K. A. Winckler B. (2019). Nestin in immature embryonic neurons affects axon growth cone morphology and semaphorin3a sensitivity. *Mol. Biol. Cell* 30 1214–1229. 10.1091/mbc.E18-06-0361 30840538 PMC6724523

[B13] Brokatzky D. Dörflinger B. Haimovici A. Weber A. Kirschnek S. Vier J. (2019). A non-death function of the mitochondrial apoptosis apparatus in immunity. *EMBO J.* 38:e100907. 10.15252/embj.2018100907 30979778 PMC6545560

[B14] Buss R. R. Sun W. Oppenheim R. W. (2006). Adaptive roles of programmed cell death during nervous system development. *Annu. Rev. Neurosci.* 29 1–35. 10.1146/annurev.neuro.29.051605.112800 16776578

[B15] Campbell D. S. Holt C. E. (2003). Apoptotic pathway and MAPKs differentially regulate chemotropic responses of retinal growth cones. *Neuron* 37 939–952. 10.1016/S0896-6273(03)00158-2 12670423

[B16] Campbell D. S. Okamoto H. (2013). Local caspase activation interacts with Slit-Robo signaling to restrict axonal arborization. *J. Cell Biol.* 203 657–672. 10.1083/jcb.201303072 24385488 PMC3840933

[B17] Carson C. Saleh M. Fung F. W. Nicholson D. W. Roskams A. J. (2005). Axonal dynactin p150Glued transports caspase-8 to drive retrograde olfactory receptor neuron apoptosis. *J. Neurosci.* 25 6092–6104. 10.1523/JNEUROSCI.0707-05.2005 15987939 PMC6725069

[B18] Cecconi F. Alvarez-Bolado G. Meyer B. I. Roth K. A. Gruss P. (1998). Apaf1 (CED-4 Homolog) regulates programmed cell death in mammalian development. *Cell* 94 727–737. 10.1016/S0092-8674(00)81732-8 9753320

[B19] Chen H. Tian J. Guo L. Du H. (2020). Caspase inhibition rescues F1Fo ATP synthase dysfunction-mediated dendritic spine elimination. *Sci. Rep.* 10:17589. 10.1038/s41598-020-74613-9 33067541 PMC7568535

[B20] Cheng X. Ferrell J. E. (2018). Apoptosis propagates through the cytoplasm as trigger waves. *Science* 361 607–612. 10.1126/science.aah4065 30093599 PMC6263143

[B21] Chipuk J. E. Moldoveanu T. Llambi F. Parsons M. J. Green D. R. (2010). The BCL-2 family reunion. *Mol. Cell* 37 299–310. 10.1016/j.molcel.2010.01.025 20159550 PMC3222298

[B22] Coleman M. L. Sahai E. A. Yeo M. Bosch M. Dewar A. Olson M. F. (2001). Membrane blebbing during apoptosis results from caspase-mediated activation of ROCK I. *Nat. Cell Biol.* 3 339–345. 10.1038/35070009 11283606

[B23] Courchet J. Lewis T. L. Lee S. Courchet V. Liou D.-Y. Aizawa S. (2013). Terminal axon branching is regulated by the LKB1-NUAK1 kinase pathway via presynaptic mitochondrial capture. *Cell* 153 1510–1525. 10.1016/j.cell.2013.05.021 23791179 PMC3729210

[B24] Cusack C. L. Swahari V. Hampton Henley W. Michael Ramsey J. Deshmukh M. (2013). Distinct pathways mediate axon degeneration during apoptosis and axon-specific pruning. *Nat. Commun.* 4:1876. 10.1038/ncomms2910 23695670 PMC4183061

[B25] D’Amelio M. Cavallucci V. Cecconi F. (2010). Neuronal caspase-3 signaling: not only cell death. *Cell Death Differ.* 17 1104–1114. 10.1038/cdd.2009.180 19960023

[B26] De León Reyes N. S. Mederos S. Varela I. Weiss L. A. Perea G. Galazo M. J. (2019). Transient callosal projections of L4 neurons are eliminated for the acquisition of local connectivity. *Nat. Commun.* 10:4549. 10.1038/s41467-019-12495-w 31591398 PMC6779895

[B27] De Maria R. Zeuner A. Eramo A. Domenichelli C. Bonci D. Grignani F. (1999). Negative regulation of erythropoiesis by caspase-mediated cleavage of GATA-1. *Nature* 401 489–493. 10.1038/46809 10519553

[B28] Deshmukh M. Johnson E. M. (1998). Evidence of a novel event during neuronal death: development of competence-to-die in response to cytoplasmic cytochrome c. *Neuron* 21 695–705. 10.1016/S0896-6273(00)80587-5 9808457

[B29] Du C. Fang M. Li Y. Li L. Wang X. (2000). Smac, a mitochondrial protein that promotes cytochrome C–dependent caspase activation by eliminating IAP inhibition. *Cell* 102 33–42. 10.1016/S0092-8674(00)00008-8 10929711

[B30] Eckelman B. P. Salvesen G. S. Scott F. L. (2006). Human inhibitor of apoptosis proteins: why XIAP is the black sheep of the family. *EMBO Rep.* 7 988–994. 10.1038/sj.embor.7400795 17016456 PMC1618369

[B31] Ellis H. M. Horvitz H. R. (1986). Genetic control of programmed cell death in the nematode C. elegans. *Cell* 44 817–829. 10.1016/0092-8674(86)90004-8 3955651

[B32] Eron S. J. Raghupathi K. Hardy J. A. (2017). Dual site phosphorylation of caspase-7 by PAK2 blocks apoptotic activity by two distinct mechanisms. *Structure* 25 27–39. 10.1016/j.str.2016.11.001 27889207 PMC5521178

[B33] Erturk A. Wang Y. Sheng M. (2014). Local pruning of dendrites and spines by caspase-3-dependent and proteasome-limited mechanisms. *J. Neurosci.* 34 1672–1688. 10.1523/JNEUROSCI.3121-13.2014 24478350 PMC6827581

[B34] Farmer T. O’Neill K. L. Naslavsky N. Luo X. Caplan S. (2019). Retromer facilitates the localization of Bcl-xL to the mitochondrial outer membrane. *Mol. Biol. Cell* 30 1138–1146. 10.1091/mbc.E19-01-0044 30840537 PMC6724524

[B35] Fogarty L. C. Song B. Suppiah Y. Hasan S. M. M. Martin H. C. Hogan S. E. (2016). Bcl-xL dependency coincides with the onset of neurogenesis in the developing mammalian spinal cord. *Mol. Cell. Neurosci.* 77 34–46. 10.1016/j.mcn.2016.09.001 27665712

[B36] Gama V. Swahari V. Schafer J. Kole A. J. Evans A. Huang Y. (2014). The E3 ligase PARC mediates the degradation of cytosolic cytochrome c to promote survival in neurons and cancer cells. *Sci. Signal.* 7:ra67. 10.1126/scisignal.2005309 25028717 PMC4182917

[B37] García M. L. Fernández A. Solas M. T. (2013). Mitochondria, motor neurons and aging. *J. Neurol. Sci.* 330 18–26. 10.1016/j.jns.2013.03.019 23628465

[B38] Garner C. C. Zhai R. G. Gundelfinger E. D. Ziv N. E. (2002). Molecular mechanisms of CNS synaptogenesis. *Trends Neurosci.* 25 243–250. 10.1016/S0166-2236(02)02152-5 11972960

[B39] Geelen J. A. G. Langman J. (1977). Closure of the neural tube in the cephalic region of the mouse embryo. *Anat. Rec.* 189 625–639. 10.1002/ar.1091890407 596653

[B40] Gibon J. Unsain N. Gamache K. Thomas R. A. De Leon A. Johnstone A. (2016). The X-linked inhibitor of apoptosis regulates long-term depression and learning rate. *FASEB J.* 30 3083–3090. 10.1096/fj.201600384R 27189977

[B41] Gilbert S. F. (2000). *Developmental Biology*, 7th Edn. Sunderland, MA: Sinauer Associates.

[B42] Giorgi C. Baldassari F. Bononi A. Bonora M. De Marchi E. Marchi S. (2012). Mitochondrial Ca2+ and apoptosis. *Cell Calcium* 52 36–43. 10.1016/j.ceca.2012.02.008 22480931 PMC3396846

[B43] Götz M. Huttner W. B. (2005). The cell biology of neurogenesis. *Nat. Rev. Mol. Cell Biol.* 6 777–788. 10.1038/nrm1739 16314867

[B44] Gu Z. Serradj N. Ueno M. Liang M. Li J. Baccei M. L. (2017). Skilled movements require non-apoptotic Bax/Bak pathway-mediated corticospinal circuit reorganization. *Neuron* 94 626–641.e4. 10.1016/j.neuron.2017.04.019 28472660 PMC5510485

[B45] Guo J. Ji Y. Ding Y. Jiang W. Sun Y. Lu B. (2016). BDNF pro-peptide regulates dendritic spines via caspase-3. *Cell Death Dis.* 7:e2264. 10.1038/cddis.2016.166 27310873 PMC5143394

[B46] Hengartner M. O. (2000). The biochemistry of apoptosis. *Nature* 407 770–776. 10.1038/35037710 11048727

[B47] Henson M. A. Tucker C. J. Zhao M. Dudek S. M. (2017). Long-term depression-associated signaling is required for an in vitro model of NMDA receptor-dependent synapse pruning. *Neurobiol. Learn. Mem.* 138 39–53. 10.1016/j.nlm.2016.10.013 27794462 PMC5336406

[B48] Houde C. Banks K. G. Coulombe N. Rasper D. Grimm E. Roy S. (2004). Caspase-7 expanded function and intrinsic expression level underlies strain-specific brain phenotype of caspase-3-null mice. *J. Neurosci.* 24 9977–9984. 10.1523/JNEUROSCI.3356-04.2004 15525783 PMC6730247

[B49] Hsu M.-T. Guo C.-L. Liou A. Y. Chang T.-Y. Ng M.-C. Florea B. I. (2015). Stage-dependent axon transport of proteasomes contributes to axon development. *Dev. Cell* 35 418–431. 10.1016/j.devcel.2015.10.018 26609957

[B50] Innocenti G. M. Price D. J. (2005). Exuberance in the development of cortical networks. *Nat. Rev. Neurosci.* 6 955–965. 10.1038/nrn1790 16288299

[B51] Jacobson M. D. Weil M. Raff M. C. (1997). Programmed cell death in animal development. *Cell* 88 347–354. 10.1016/S0092-8674(00)81873-5 9039261

[B52] Jiao S. Li Z. (2011). Nonapoptotic function of BAD and BAX in long-term depression of synaptic transmission. *Neuron* 70 758–772. 10.1016/j.neuron.2011.04.004 21609830 PMC3102234

[B53] Joazeiro C. A. P. Weissman A. M. (2000). RING finger proteins: mediators of ubiquitin ligase activity. *Cell* 102 549–552. 10.1016/S0092-8674(00)00077-5 11007473

[B54] Julien O. Wells J. A. (2017). Caspases and their substrates. *Cell Death Differ.* 24 1380–1389. 10.1038/cdd.2017.44 28498362 PMC5520456

[B55] Kale J. Osterlund E. J. Andrews D. W. (2018). BCL-2 family proteins: changing partners in the dance towards death. *Cell Death Differ.* 25 65–80. 10.1038/cdd.2017.186 29149100 PMC5729540

[B56] Karch J. Kwong J. Q. Burr A. R. Sargent M. A. Elrod J. W. Peixoto P. M. (2013). Bax and Bak function as the outer membrane component of the mitochondrial permeability pore in regulating necrotic cell death in mice. *eLife* 2:e00772. 10.7554/eLife.00772 23991283 PMC3755340

[B57] Karch J. Schips T. G. Maliken B. D. Brody M. J. Sargent M. A. Kanisicak O. (2017). Autophagic cell death is dependent on lysosomal membrane permeability through Bax and Bak. *eLife* 6:e30543. 10.7554/eLife.30543 29148970 PMC5697932

[B58] Katow H. Kanaya T. Ogawa T. Egawa R. Yawo H. (2017). Regulation of axon arborization pattern in the developing chick ciliary ganglion: possible involvement of caspase 3. *Dev. Growth Differ.* 59 115–128. 10.1111/dgd.12346 28430358

[B59] Kawai K. Itoh T. Itoh A. Horiuchi M. Wakayama K. Bannerman P. (2009). Maintenance of the relative proportion of oligodendrocytes to axons even in the absence of BAX and BAK. *Eur. J. Neurosci.* 30 2030–2041. 10.1111/j.1460-9568.2009.06988.x 20128842 PMC2830116

[B60] Ke F. F. S. Vanyai H. K. Cowan A. D. Delbridge A. R. D. Whitehead L. Grabow S. (2018). Embryogenesis and adult life in the absence of intrinsic apoptosis effectors BAX, BAK, and BOK. *Cell* 173 1217–1230.e17. 10.1016/j.cell.2018.04.036 29775594

[B61] Khatri N. Gilbert J. P. Huo Y. Sharaflari R. Nee M. Qiao H. (2018). The autism protein Ube3A/E6AP remodels neuronal dendritic arborization via caspase-dependent microtubule destabilization. *J. Neurosci.* 38 363–378. 10.1523/JNEUROSCI.1511-17.2017 29175955 PMC5761614

[B62] Knudson C. M. Tung K. S. K. Tourtellotte W. G. Brown G. A. J. Korsmeyer S. J. (1995). Bax-Deficient mice with lymphoid hyperplasia and male germ cell death. *Science* 270 96–99. 10.1126/science.270.5233.96 7569956

[B63] Kuida K. Haydar T. F. Kuan C.-Y. Gu Y. Taya C. Karasuyama H. (1998). Reduced apoptosis and cytochrome c–mediated caspase activation in mice lacking caspase 9. *Cell* 94 325–337. 10.1016/S0092-8674(00)81476-2 9708735

[B64] Kuida K. Zheng T. S. Na S. Kuan C.-Y. Yang D. Karasuyama H. (1996). Decreased apoptosis in the brain and premature lethality in CPP32-deficient mice. *Nature* 384 368–372. 10.1038/384368a0 8934524

[B65] Kumar S. (2007). Caspase function in programmed cell death. *Cell Death Differ.* 14 32–43. 10.1038/sj.cdd.4402060 17082813

[B66] Kuranaga E. Miura M. (2007). Nonapoptotic functions of caspases: caspases as regulatory molecules for immunity and cell-fate determination. *Trends Cell Biol.* 17 135–144. 10.1016/j.tcb.2007.01.001 17275304

[B67] Lakhani S. A. (2006). Caspases 3 and 7: key mediators of mitochondrial events of apoptosis. *Science* 311 847–851. 10.1126/science.1115035 16469926 PMC3738210

[B68] Lartigue L. Medina C. Schembri L. Chabert P. Zanese M. Tomasello F. (2008). An intracellular wave of cytochrome c propagates and precedes Bax redistribution during apoptosis. *J. Cell Sci.* 121 3515–3523. 10.1242/jcs.029587 18840646

[B69] Ledonne F. Orduz D. Mercier J. Vigier L. Grove E. A. Tissir F. (2016). Targeted inactivation of Bax reveals a subtype-specific mechanism of cajal-retzius neuron death in the postnatal cerebral cortex. *Cell Rep.* 17 3133–3141. 10.1016/j.celrep.2016.11.074 28009284

[B70] Leonard J. R. Klocke B. J. D’sa C. Flavell R. A. Roth K. A. (2002). Strain-Dependent neurodevelopmental abnormalities in caspase-3-deficient mice. *J. Neuropathol. Exp. Neurol.* 61 673–677. 10.1093/jnen/61.8.673 12152782

[B71] Li H. Chen Y. Jones A. F. Sanger R. H. Collis L. P. Flannery R. (2008). Bcl-xL induces Drp1-dependent synapse formation in cultured hippocampal neurons. *Proc. Natl. Acad. Sci. U.S.A.* 105 2169–2174. 10.1073/pnas.0711647105 18250306 PMC2542873

[B72] Li J. Yuan J. (2008). Caspases in apoptosis and beyond. *Oncogene* 27 6194–6206. 10.1038/onc.2008.297 18931687

[B73] Li K. Li Y. Shelton J. M. Richardson J. A. Spencer E. Chen Z. J. (2000). Cytochrome c deficiency causes embryonic lethality and attenuates stress-induced apoptosis. *Cell* 101 389–399. 10.1016/S0092-8674(00)80849-1 10830166

[B74] Li Z. Jo J. Jia J.-M. Lo S.-C. Whitcomb D. J. Jiao S. (2010). Caspase-3 activation via mitochondria is required for long-term depression and AMPA receptor internalization. *Cell* 141 859–871. 10.1016/j.cell.2010.03.053 20510932 PMC2909748

[B75] Lindsten T. Ross A. J. King A. Zong W.-X. Rathmell J. C. Shiels H. A. (2000). The combined functions of proapoptotic Bcl-2 family members Bak and Bax are essential for normal development of multiple tissues. *Mol. Cell* 6 1389–1399. 10.1016/s1097-2765(00)00136-2 11163212 PMC3057227

[B76] Liu X. Zhang Q. Jiang Q. Bai B. Du X. Wang F. (2018). Genetic screening and functional analysis of CASP9 mutations in a Chinese cohort with neural tube defects. *CNS Neurosci. Ther.* 24 394–403. 10.1111/cns.12797 29365368 PMC6489964

[B77] Lo S.-C. Wang Y. Weber M. Larson J. L. Scearce-Levie K. Sheng M. (2015). Caspase-3 deficiency results in disrupted synaptic homeostasis and impaired attention control. *J. Neurosci.* 35 2118–2132. 10.1523/JNEUROSCI.3280-14.2015 25653368 PMC6705356

[B78] Martinon F. Tschopp J. (2004). Inflammatory caspases: linking an intracellular innate immune system to autoinflammatory diseases. *Cell* 117 561–574. 10.1016/j.cell.2004.05.004 15163405

[B79] Mashima T. Naito M. Tsuruo T. (1999). Caspase-mediated cleavage of cytoskeletal actin plays a positive role in the process of morphological apoptosis. *Oncogene* 18 2423–2430. 10.1038/sj.onc.1202558 10229193

[B80] Massa V. Savery D. Ybot-Gonzalez P. Ferraro E. Rongvaux A. Cecconi F. (2009). Apoptosis is not required for mammalian neural tube closure. *Proc. Natl. Acad. Sci. U.S.A.* 106 8233–8238. 10.1073/pnas.0900333106 19420217 PMC2688898

[B81] Michaelidis T. M. Sendtner M. Cooper J. D. Airaksinen M. S. Holtmann B. Meyer M. (1996). Inactivation of bcl-2 results in progressive degeneration of motoneurons, sympathetic and sensory neurons during early postnatal development. *Neuron* 17 75–89. 10.1016/S0896-6273(00)80282-2 8755480

[B82] Motoyama N. Wang F. Roth K. Sawa H. Nakayama K. Nakayama K. (1995). Massive cell death of immature hematopoietic cells and neurons in Bcl-x-deficient mice. *Science* 267 1506–1510. 10.1126/science.7878471 7878471

[B83] Mukherjee A. Williams D. W. (2017). More alive than dead: non-apoptotic roles for caspases in neuronal development, plasticity and disease. *Cell Death Differ.* 24 1411–1421. 10.1038/cdd.2017.64 28644437 PMC5520460

[B84] Nakamura A. Swahari V. Plestant C. Smith I. McCoy E. Smith S. (2016). Bcl-xL is essential for the survival and function of differentiated neurons in the cortex that control complex behaviors. *J. Neurosci.* 36 5448–5461. 10.1523/JNEUROSCI.4247-15.2016 27194326 PMC4871982

[B85] Neuspiel M. Schauss A. C. Braschi E. Zunino R. Rippstein P. Rachubinski R. A. (2008). Cargo-selected transport from the mitochondria to peroxisomes is mediated by vesicular carriers. *Curr. Biol.* 18 102–108. 10.1016/j.cub.2007.12.038 18207745

[B86] Nikolaev A. McLaughlin T. O’Leary D. D. M. Tessier-Lavigne M. (2009). APP binds DR6 to trigger axon pruning and neuron death via distinct caspases. *Nature* 457 981–989. 10.1038/nature07767 19225519 PMC2677572

[B87] Nonomura K. Yamaguchi Y. Hamachi M. Koike M. Uchiyama Y. Nakazato K. (2013). Local apoptosis modulates early mammalian brain development through the elimination of morphogen-producing cells. *Dev. Cell* 27 621–634. 10.1016/j.devcel.2013.11.015 24369835

[B88] Ohsawa S. Hamada S. Kuida K. Yoshida H. Igaki T. Miura M. (2010). Maturation of the olfactory sensory neurons by Apaf-1/caspase-9-mediated caspase activity. *Proc. Natl. Acad. Sci. U.S.A.* 107 13366–13371. 10.1073/pnas.0910488107 20624980 PMC2922127

[B89] Otero M. G. Alloatti M. Cromberg L. E. Almenar-Queralt A. Encalada S. E. Devoto V. M. P. (2014). Fast axonal transport of the proteasome complex depends on membrane interaction and molecular motor function. *J. Cell Sci.* 127 1537–1549. 10.1242/jcs.140780 24522182

[B90] Pacheco A. Gallo G. (2016). Actin filament-microtubule interactions in axon initiation and branching. *Brain Res. Bull.* 126 300–310. 10.1016/j.brainresbull.2016.07.013 27491623 PMC5518172

[B91] Pacher P. Hajnóczky G. (2001). Propagation of the apoptotic signal by mitochondrial waves. *EMBO J.* 20 4107–4121. 10.1093/emboj/20.15.4107 11483514 PMC149166

[B92] Paridaen J. T. Huttner W. B. (2014). Neurogenesis during development of the vertebrate central nervous system. *EMBO Rep.* 15 351–364. 10.1002/embr.201438447 24639559 PMC3989667

[B93] Parrish A. B. Freel C. D. Kornbluth S. (2013). Cellular mechanisms controlling caspase activation and function. *Cold Spring Harb. Perspect. Biol.* 5:a008672. 10.1101/cshperspect.a008672 23732469 PMC3660825

[B94] Péquignot M. O. Provost A. C. Sallé S. Taupin P. Sainton K. M. Marchant D. (2003). Major role of BAX in apoptosis during retinal development and in establishment of a functional postnatal retina. *Dev. Dyn.* 228 231–238. 10.1002/dvdy.10376 14517994

[B95] Petros A. M. Olejniczak E. T. Fesik S. W. (2004). Structural biology of the Bcl-2 family of proteins. *Biochim. Biophys. Acta* 1644 83–94. 10.1016/j.bbamcr.2003.08.012 14996493

[B96] Pinan-Lucarre B. Gabel C. V. Reina C. P. Hulme S. E. Shevkoplyas S. S. Slone R. D. (2012). The core apoptotic executioner proteins CED-3 and CED-4 promote initiation of neuronal regeneration in *Caenorhabditis elegans*. *PLoS Biol.* 10:e1001331. 10.1371/journal.pbio.1001331 22629231 PMC3358320

[B97] Purves D. Augustine G. J. Fitzpatrick D. Hall W. C. LaMantia A.-S. Moone R. D. (2018). *Neuroscience*. New York, NY: Oxford University Press.

[B98] Raina D. Pandey P. Ahmad R. Bharti A. Ren J. Kharbanda S. (2005). c-Abl tyrosine kinase regulates caspase-9 autocleavage in the apoptotic response to DNA damage^∗^. *J. Biol. Chem.* 280 11147–11151. 10.1074/jbc.M413787200 15657060

[B99] Raz V. Carlotti F. Vermolen B. J. van der Poel E. Sloos W. C. R. Knaän-Shanzer S. (2006). Changes in lamina structure are followed by spatial reorganization of heterochromatic regions in caspase-8-activated human mesenchymal stem cells. *J. Cell Sci.* 119 4247–4256. 10.1242/jcs.03180 17003109

[B100] Ribeil J.-A. Zermati Y. Vandekerckhove J. Cathelin S. Kersual J. Dussiot M. (2007). Hsp70 regulates erythropoiesis by preventing caspase-3-mediated cleavage of GATA-1. *Nature* 445 102–105. 10.1038/nature05378 17167422

[B101] Rodriguez J. Lazebnik Y. (1999). Caspase-9 and APAF-1 form an active holoenzyme. *Genes Dev.* 13 3179–3184. 10.1101/gad.13.24.3179 10617566 PMC317200

[B102] Sakamaki K. Inoue T. Asano M. Sudo K. Kazama H. Sakagami J. (2002). Ex vivo whole-embryo culture of caspase-8-deficient embryos normalize their aberrant phenotypes in the developing neural tube and heart. *Cell Death Differ.* 9 1196–1206. 10.1038/sj.cdd.4401090 12404118

[B103] Scorrano L. Oakes S. A. Opferman J. T. Cheng E. H. Sorcinelli M. D. Pozzan T. (2003). BAX and BAK regulation of endoplasmic reticulum Ca2+: a control point for apoptosis. *Science* 300 135–139. 10.1126/science.1081208 12624178

[B104] Serrano B. P. Hardy J. A. (2018). Phosphorylation by protein kinase A disassembles the caspase-9 core. *Cell Death Differ.* 25 1025–1039. 10.1038/s41418-017-0052-9 29352269 PMC5988757

[B105] Serrano B. P. Szydlo H. S. Alfandari D. Hardy J. A. (2017). Active site–adjacent phosphorylation at Tyr-397 by c-Abl kinase inactivates caspase-9. *J. Biol. Chem.* 292 21352–21365. 10.1074/jbc.M117.811976 29066624 PMC5766954

[B106] Shalini S. Dorstyn L. Dawar S. Kumar S. (2015). Old, new and emerging functions of caspases. *Cell Death Differ.* 22 526–539. 10.1038/cdd.2014.216 25526085 PMC4356345

[B107] Simon D. J. Weimer R. M. McLaughlin T. Kallop D. Stanger K. Yang J. (2012). A caspase cascade regulating developmental axon degeneration. *J. Neurosci.* 32 17540–17553. 10.1523/JNEUROSCI.3012-12.2012 23223278 PMC3532512

[B108] Singh R. Letai A. Sarosiek K. (2019). Regulation of apoptosis in health and disease: the balancing act of BCL-2 family proteins. *Nat. Rev. Mol. Cell Biol.* 20 175–193. 10.1038/s41580-018-0089-8 30655609 PMC7325303

[B109] Slee E. A. Adrain C. Martin S. J. (2001). Executioner caspase-3, -6, and -7 perform distinct, non-redundant roles during the demolition phase of apoptosis^∗^. *J. Biol. Chem.* 276 7320–7326. 10.1074/jbc.M008363200 11058599

[B110] Smith J. L. Schoenwolf G. C. (1997). Neurulation: coming to closure. *Trends Neurosci.* 20 510–517. 10.1016/S0166-2236(97)01121-1 9364665

[B111] Sokolowski J. D. Gamage K. K. Heffron D. S. LeBlanc A. C. Deppmann C. D. Mandell J. W. (2014). Caspase-mediated cleavage of actin and tubulin is a common feature and sensitive marker of axonal degeneration in neural development and injury. *Acta Neuropathol. Commun.* 2:16. 10.1186/2051-5960-2-16 24507707 PMC3996144

[B112] Spellicy C. J. Norris J. Bend R. Bupp C. Mester P. Reynolds T. (2018). Key apoptotic genes APAF1 and CASP9 implicated in recurrent folate-resistant neural tube defects. *Eur. J. Hum. Genet.* 26 420–427. 10.1038/s41431-017-0025-y 29358613 PMC5838979

[B113] Stiles J. Jernigan T. L. (2010). The basics of brain development. *Neuropsychol. Rev.* 20 327–348. 10.1007/s11065-010-9148-4 21042938 PMC2989000

[B114] Suzanne M. Steller H. (2013). Shaping organisms with apoptosis. *Cell Death Differ.* 20 669–675. 10.1038/cdd.2013.11 23449394 PMC3619238

[B115] Tait S. W. G. Parsons M. J. Llambi F. Bouchier-Hayes L. Connell S. Muñoz-Pinedo C. (2010). Resistance to caspase-independent cell death requires persistence of intact mitochondria. *Dev. Cell* 18 802–813. 10.1016/j.devcel.2010.03.014 20493813 PMC3004027

[B116] Taylor R. C. Cullen S. P. Martin S. J. (2008). Apoptosis: controlled demolition at the cellular level. *Nat. Rev. Mol. Cell Biol.* 9 231–241. 10.1038/nrm2312 18073771

[B117] Unsain N. Higgins J. M. Parker K. N. Johnstone A. D. Barker P. A. (2013). XIAP regulates caspase activity in degenerating axons. *Cell Rep.* 4 751–763. 10.1016/j.celrep.2013.07.015 23954782

[B118] Uribe V. Wong B. K. Y. Graham R. K. Cusack C. L. Skotte N. H. Pouladi M. A. (2012). Rescue from excitotoxicity and axonal degeneration accompanied by age-dependent behavioral and neuroanatomical alterations in caspase-6-deficient mice. *Hum. Mol. Genet.* 21 1954–1967. 10.1093/hmg/dds005 22262731 PMC3315204

[B119] Varfolomeev E. E. Schuchmann M. Luria V. Chiannilkulchai N. Beckmann J. S. Mett I. L. (1998). Targeted disruption of the mouse caspase 8 gene ablates cell death induction by the TNF receptors, Fas/Apo1, and DR3 and is lethal prenatally. *Immunity* 9 267–276. 10.1016/S1074-7613(00)80609-3 9729047

[B120] Verma P. (2005). Axonal protein synthesis and degradation are necessary for efficient growth cone regeneration. *J. Neurosci.* 25 331–342. 10.1523/JNEUROSCI.3073-04.2005 15647476 PMC3687202

[B121] Voss O. H. Kim S. Wewers M. D. Doseff A. I. (2005). Regulation of monocyte apoptosis by the protein kinase cδ-dependent phosphorylation of caspase-3^∗^. *J. Biol. Chem.* 280 17371–17379. 10.1074/jbc.M412449200 15716280

[B122] Walsh J. G. Cullen S. P. Sheridan C. Lüthi A. U. Gerner C. Martin S. J. (2008). Executioner caspase-3 and caspase-7 are functionally distinct proteases. *Proc. Natl. Acad. Sci. U.S.A.* 105 12815–12819. 10.1073/pnas.0707715105 18723680 PMC2529079

[B123] Wang G. Sun L. Reina C. P. Song I. Gabel C. V. Driscoll M. (2019). CED-4 CARD domain residues can modulate non-apoptotic neuronal regeneration functions independently from apoptosis. *Sci. Rep.* 9:13315. 10.1038/s41598-019-49633-9 31527664 PMC6746752

[B124] Wang J.-Y. Chen F. Fu X.-Q. Ding C.-S. Zhou L. Zhang X.-H. (2014). Caspase-3 cleavage of dishevelled induces elimination of postsynaptic structures. *Dev. Cell* 28 670–684. 10.1016/j.devcel.2014.02.009 24631402

[B125] Washausen S. Scheffel T. Brunnett G. Knabe W. (2018). Possibilities and limitations of three-dimensional reconstruction and simulation techniques to identify patterns, rhythms and functions of apoptosis in the early developing neural tube. *Hist. Philos. Life Sci.* 40:55. 10.1007/s40656-018-0222-1 30159859

[B126] Weghorst F. Mirzakhanyan Y. Samimi K. Dhillon M. Barzik M. Cunningham L. L. (2020). Caspase-3 cleaves extracellular vesicle proteins during auditory brainstem development. *Front. Cell. Neurosci.* 14:573345. 10.3389/fncel.2020.573345 33281555 PMC7689216

[B127] Weil M. Jacobson M. D. Raff M. C. (1997). Is programmed cell death required for neural tube closure? *Curr. Biol.* 7 281–284. 10.1016/S0960-9822(06)00125-4 9094312

[B128] Westphal D. Sytnyk V. Schachner M. Leshchyns’ka I. (2010). Clustering of the neural cell adhesion molecule (NCAM) at the neuronal cell surface induces caspase-8- and -3-dependent changes of the spectrin meshwork required for NCAM-mediated neurite outgrowth. *J. Biol. Chem.* 285 42046–42057. 10.1074/jbc.M110.177147 20961848 PMC3009930

[B129] Wickman G. Julian L. Olson M. F. (2012). How apoptotic cells aid in the removal of their own cold dead bodies. *Cell Death Differ.* 19 735–742. 10.1038/cdd.2012.25 22421963 PMC3321633

[B130] Yamaguchi Y. Miura M. (2015). Programmed cell death in neurodevelopment. *Dev. Cell* 32 478–490. 10.1016/j.devcel.2015.01.019 25710534

[B131] Yoshida H. Kong Y.-Y. Yoshida R. Elia A. J. Hakem A. Hakem R. (1998). Apaf1 is required for mitochondrial pathways of apoptosis and brain development. *Cell* 94 739–750. 10.1016/S0092-8674(00)81733-X 9753321

[B132] Youle R. J. Strasser A. (2008). The BCL-2 protein family: opposing activities that mediate cell death. *Nat. Rev. Mol. Cell Biol.* 9 47–59. 10.1038/nrm2308 18097445

[B133] Yuan J. Yankner B. A. (2000). Apoptosis in the nervous system. *Nature* 407 802–809. 10.1038/35037739 11048732

[B134] Yuan J. Shaham S. Ledoux S. Ellis H. M. Horvitz H. R. (1993). The C. elegans cell death gene ced-3 encodes a protein similar to mammalian interleukin-1β-converting enzyme. *Cell* 75 641–652. 10.1016/0092-8674(93)90485-9 8242740

[B135] Zamaraev A. V. Kopeina G. S. Prokhorova E. A. Zhivotovsky B. Lavrik I. N. (2017). Post-translational modification of caspases: the other side of apoptosis regulation. *Trends Cell Biol.* 27 322–339. 10.1016/j.tcb.2017.01.003 28188028

[B136] Zhang D. Armstrong J. S. (2007). Bax and the mitochondrial permeability transition cooperate in the release of cytochrome c during endoplasmic reticulum-stress-induced apoptosis. *Cell Death Differ.* 14 703–715. 10.1038/sj.cdd.4402072 17170750

[B137] Zhang J. Webster J. D. Dugger D. L. Goncharov T. Roose-Girma M. Hung J. (2019). Ubiquitin ligases cIAP1 and cIAP2 limit cell death to prevent inflammation. *Cell Rep.* 27 2679–2689.e3. 10.1016/j.celrep.2019.04.111 31141691

[B138] Zhang M. Zheng J. Nussinov R. Ma B. (2017). Release of cytochrome C from Bax pores at the mitochondrial membrane. *Sci. Rep.* 7:2635. 10.1038/s41598-017-02825-7 28572603 PMC5453941

[B139] Zhou X. Zeng W. Li H. Chen H. Wei G. Yang X. (2018). Rare mutations in apoptosis related genes APAF1, CASP9, and CASP3 contribute to human neural tube defects. *Cell Death Dis.* 9:43. 10.1038/s41419-017-0096-2 29352212 PMC5833651

[B140] Ziegler D. V. Vindrieux D. Goehrig D. Jaber S. Collin G. Griveau A. (2021). Calcium channel ITPR2 and mitochondria–ER contacts promote cellular senescence and aging. *Nat. Commun.* 12:720. 10.1038/s41467-021-20993-z 33526781 PMC7851384

